# Pharmacodynamics in hypothermia

**DOI:** 10.1186/cc11264

**Published:** 2012-06-07

**Authors:** Torkjel Tveita

**Affiliations:** 1 Anesthesia and Critical Care, Institute of Clinical Medicine, University of Tromsø, Norway; 2 University Hospital of Northern Norway, Tromsø, Norway

## Introduction

Guidelines for using inotropic drugs to support cardiovascular function at low core temperatures are not well characterized. The safe application of inotropic drugs during normothermic conditions, routinely used to treat cardiovascular instability by effectively increasing cardiac output (CO) and improve end-organ perfusion associated with acute heart failure [1], is based on a detailed understanding of pharmacodynamics and pharmacokinetics of these drugs. Detailed knowledge of temperature-dependent changes in pharmacodynamics and pharmacokinetics of such cardioactive drugs is essential for establishing treatment guidelines.

## Therapeutic hypothermia

Over the last decade therapeutic hypothermia has been established as a recognized intervention to increase survival and improve neurologic outcome in adult comatose cardiac arrest survivors [2-6]. However, after return of spontaneous circulation (ROSC) and coronary revascularization, more than 50% of survivors suffer from acute heart failure and need inotropic cardiac support to resume adequate circulatory function [2] after induction of hypothermia when the core temperature is deliberately lowered to 34 to 32ºC and maintained for 24 to 48 hours. In contrast, in patients hospitalized for acute heart failure without hypothermia a subgroup of about only 10% received inotropic drugs [7].

## Accidental hypothermia

Another group of patients in need of inotropic drug therapy at low core temperature are accidental hypothermia patients displaying hypothermia-induced cardiac failure during rewarming, ranging from mild reduction of CO to the fulminant circulatory shock termed rewarming shock [8-11]. Rewarming shock is a clinically descriptive term that refers to a pathophysiologic state of cardiovascular collapse taking place during or after rewarming from accidental hypothermia [12], recognized as a progressive reduction of CO and a sudden fall in arterial blood pressure. In order to treat or prevent rewarming shock, cardioactive inotropic drugs are commonly necessary to elevate a low CO.

Research in experimental hypothermia has displayed a substantial depression of LV myocardial function in earlier studies [8,11,13] as well as in recent studies [9,13,14]. Based on the results from these studies, cellular calcium overload, disturbed calcium homeostasis, changes in myocardial myofilament responsiveness to intracellular calcium as well as impaired high-energy phosphate homeostasis could all be proposed as important factors leading to the changes observed in the hypothermic heart and contributing to failure of functional recovery during rewarming [15-18].

## β-receptor agonists

In the acutely failing heart postoperatively, only drugs such as epinephrine and NE provide positive inotropy and perfusion pressure. Epinephrine acts through stimulation via sarcolemmal β-adrenoceptors, causing phosphorylation of the sarcolemmal L-type Ca^2+ ^channel via cyclic AMP and protein kinase A pathways. This phosphorylation increases the open probability of the channel [19], allowing for greater trans-sarcolemmal Ca^2+ ^influx with each depolarization and producing, in part, the positive inotropic effect of epinephrine.

## Vascular effects

Only a few experiments studying effects of epinephrine during hypothermia or/and rewarming using *in vivo *animal models have been published. Some authors report that both β_1_-adrenoceptors and α-adrenoceptors increase their sensitivity to catecholamines during hypothermia [18,20-22] as β_1_-adrenoceptor activity was potentiated by low temperature, and they claim the existence of hypothermia-induced supersensitivity and increased agonist activity for β_1_-adrenoceptors. In support of this view, a left shift of the concentration-response curve for epinephrine during hypothermia has been reported [23]. However, others suggest hypothermia-induced increase in sensitivity for both α_1_-adrenoceptors and α_2_-adrenoceptors during cooling [24], but that sensitivity of β_1_-adrenoceptors is not increased to the same extent as α-adrenoceptors. In contrast, other researchers have reported a hypothermia-induced supersensitivity of β_1_-adrenoceptors [18,20,21]. Rubinstein reported that hypothermia modified the vascular response to epinephrine [25]; that is, the epinephrine doses that induced vasodilation during normothermic conditions increased TPR at 25°C. He also claimed that myocardial contractile effects of epinephrine is reduced at low temperatures (covered below), a view also supported by others [25,25-27]. Experimental data show that the sympathetic nervous system could be switched off at a threshold temperature about 29°C and hypotensive patients with temperatures below this may benefit from infusions of exogenous catecholamines [28]. In addition, if CO could be elevated pharmacologically, rewarming by any means becomes more efficient [17,29]. Some researchers recommend infusion of low doses of catecholamines in patients who have lower blood pressure than would be expected for that degree of hypothermia and who are not responding to crystalloids and rewarming [29].

It therefore seems that the use of vasoactive drugs during hypothermic conditions remains quite contradictory.

## Cardiac effects

Some sources claim that the hypothermic heart is unresponsive [30] or little responsive [31] to cardioactive drugs, and the last reference as well as recommendation of the American Heart Association [26] refer to the potential hazard of overmedication, due to delayed drug metabolism leading to accumulation to toxic levels in patients suffering from deep or severe hypothermia, if used repeatedly. The AHA recommends the following algorithm for treatment of hypothermia: below 30°C i.v. epinephrine should not be given, but above 30°C epinephrine should be given, if indicated, but at longer than standard intervals. To date, there are no prospective clinical studies to support the recommendation to avoid epinephrine during hypothermic CPR, but a preclinical work report major side effects of repeated epinephrine administration during experimental hypothermic CPR in pigs [32]. Even in the last recommendations from the AHA it was stated that treatment of severe hypothermia (temperature <30°C) in the field remains controversial [26].

A reduction of CO and SV by inducing varying levels of hypercalcaemia during hypothermia (28ºC) was reported by Schiffmann and colleagues [18]. Following infusion of epinephrine during these experimental conditions, CO and SV were even more depressed [18]. These findings shear similarities with findings in our intact animal models: infusion of epinephrine, which theoretically will induce opening of Ca^2+ ^channels, increase calcium influx and elevate intracellular calcium even further, caused a significant depression of myocardial function during hypothermic as well as post-hypothermic conditions [13,33,34]. This made us conclude that hypothermia and rewarming may cause alterations in the pharmacodynamic effects of α-receptor and β-receptor mediated drugs [13,33,35-37] or induce changes in receptor affinity for these drugs. Further, we found that low-dose epinephrine managed to maintain positive inotropic effects on LV cardiac function during cooling to 30ºC, but these effects vanished during cooling to 28ºC. If the epinephrine dose was increased at these low temperatures SV and CO were not elevated but a significant increase in afterload took place. Thus we conclude that if epinephrine is applied during hypothermia the therapeutic window appears narrow with short distance to unwanted side effects. Further, the prehypothermic dose-dependent increase in LV function in response to epinephrine was not present after rewarming in the group that had received epinephrine during hypothermia [13,33,35,38], and if epinephrine was infused during rewarming vascular side effects of epinephrine (vasoconstriction) dominated [13,38] without elevating CO above pre-hypothermic control values at any temperature.

During cooling there is a reduction in Ca^2+ ^sensitivity of troponin C due to protein kinase A (PKA)-induced phosphorylation of troponin I [39], in addition to the hypothermia-induced calcium overload [18,19]. Further, it is documented that PKA may inhibit the activity of adenylyl cyclase (AC) 5 [40,41], predominantly expressed in the heart [42], which catalyzes ATP to cAMP. Based on this information, it is possible that lack of positive inotropic effect on cardiac function of β-receptor stimulation during hypothermia may be due to the inhibitory effects on AC 5 activity of PKA phosphorylation and/or a potential desensitization and internalization of the β-adrenoceptor. Another way of interpreting these results is that the efficacy of signal transduction through G-protein coupled receptors is rapidly decreased through mechanisms like desensitization and internalization, mechanisms that will avoid receptor overstimulation. However, during pathologic conditions, like acute heart failure following acute coronary syndromes and cardiac standstill, or as a consequence of exposure to accidental hypothermia, it is necessary for cardiac myocytes to produce cAMP over the limitation of such adaptations.

## Dopamine

The still widespread use of DA in perioperative and intensive care, the explicit recommendation for its use in accidental hypothermia guidelines, and possible positive effects in hypothermia as reported from experimental studies all need evaluation. From experimental hypothermia research we found that DA improved both systolic and diastolic function in hypothermia [43]. However, at 25°C no beneficial effect was seen on CI as SI decreased with incrementing DA dosages. Increased SVRI at high-dose DA at 25°C suggests α-adrenergic involvement not seen in normothermia. Properties of the low-flow, high-viscosity circulatory state, combined with serious alterations in the pharmacokinetics of DA, may explain lack of beneficial - and potentially harmful - effects from DA administration at 25°C.

## Milrinone

Milrinone is a phosphodiesterase (PDE) 3 inhibitor that is dominantly expressed in the heart and vascular tissues. The site of action of milrinone is cytosolic, and the administration of a PDE3 inhibitor increases cAMP only in the cardiovascular system, which subsequently enhance cardiac contraction and induce vasodilation during normothermic conditions [44]. In a recent experiment [45], using milrinone as a model drug of intracellular mode of action, the positive cardiac effects of this drug was demonstrated during normothermic conditions (30% increase in SV and CO) and remained during cooling to 15ºC. These effects of milrinone on cardiac function stay in essential contrast to those in our previous studies testing effects of α-receptor and β-receptor mediated drugs [35,38] during cooling and rewarming.

## Conclusion

Taken together, the use of cardioactive drugs during hypothermic conditions remains quite contradictory. Therefore, pharmacologic treatment applied during the clinically challenging modalities, therapeutic as well as accidental hypothermia, call upon written treatment protocols or guidelines that are so far largely missing or at least not properly updated. More research is needed to explore temperature-dependent changes in pharmacodynamics and pharmacokinetics of cardioactive drugs to write these guidelines. Thus, due to significant hypothermia-induced alterations of cardiac as well as vascular adrenoceptor sensitivity, the use of cardioactive agents not affecting these receptor systems are advised during hypothermic conditions.
